# The genome sequence of the northern pintail, *Anas acuta *Linnaeus, 1758

**DOI:** 10.12688/wellcomeopenres.22770.1

**Published:** 2024-08-12

**Authors:** Michelle F. O’Brien, Rosa Lopez Colom, Natural History Museum Genome Acquisition Lab, Wellcome Sanger Institute Tree of Life Management, Samples and Laboratory team, Wellcome Sanger Institute Scientific Operations: Sequencing Operations, Wellcome Sanger Institute Tree of Life Core Informatics team, Tree of Life Core Informatics collective, Darwin Tree of Life Consortium

**Affiliations:** 1 Wildfowl & Wetlands Trust, Slimbridge, England, UK

**Keywords:** Anas acuta, northern pintail, genome sequence, chromosomal, Anseriformes

## Abstract

We present a genome assembly from an individual female *Anas acuta* (the northern pintail; Chordata; Aves; Anseriformes; Anatidae). The genome sequence spans 1,189.30 megabases. Most of the assembly is scaffolded into 39 chromosomal pseudomolecules, including the W and Z sex chromosomes. The mitochondrial genome has also been assembled and is 16.6 kilobases in length.

## Species taxonomy

Eukaryota; Opisthokonta; Metazoa; Eumetazoa; Bilateria; Deuterostomia; Chordata; Craniata; Vertebrata; Gnathostomata; Teleostomi; Euteleostomi; Sarcopterygii; Dipnotetrapodomorpha; Tetrapoda; Amniota; Sauropsida; Sauria; Archelosauria; Archosauria; Dinosauria; Saurischia; Theropoda; Coelurosauria; Aves; Neognathae; Galloanserae; Anseriformes; Anatidae; Anatinae; *Anas*; *Anas acuta* Linnaeus, 1758 (NCBI:txid28680).

## Background

The Northern Pintail ( *Anas acuta*) is a medium size duck weighing up to 1300 g, with a length of up to 66 cm. This is considered to be a particularly elegant species with a slim neck and relatively long bill. It is named for the long tail feathers of the male which extend to a point ( Gooders & Boyer, 1986).

The male has a chocolate brown head and neck with a white underside extending down to cover the chest area and white lines that curve round to the side of the head. The rest of the feathers are grey overall with a black rear end and part of the wings. The female is a more muted colour with spotted feathers and an overall brown/grey camouflage ( Gooders & Boyer, 1986).

This is a species of dabbling duck and able to feed in water depths up to 30 cm. They feed on both vegetable matter and animate matter depending on the time of year ( Gooders & Boyer, 1986). Their longer necks allow them to exploit feeding areas inaccessible to some other duck species ( Owen, 1977).

Northern pintails breed over large areas of the northern Holarctic, North America and Eurasia. This species is migratory and in most regions is a long-distance migrant. Its wintering areas are spread across western and southern Europe, Africa, Asia, India, China and Japan. In North America they move south and leave most of their breeding range during winter ( Delany *et al.*, 2006). They lay 7 to 9 eggs in shallow ground nests lined with down and incubation takes up to 24 days ( Gooders & Boyer, 1986). *A*. *acuta* can live up to 29.5 years old ( Fransson *et al.*, 2023).

Northern pintails have been shown to carry out intercontinental migrations between Asia and North America and homogenous population genetic data between North America and Asia indicates genetic flow ( Flint *et al.*, 2009; Miller *et al.*, 2005; Nicolai *et al.*, 2005). Phylogenetic analysis of low pathogenic avian influenza (LPAI) viruses found in swabs taken from Northern pintails in Alaska have detected as many as four gene segments of Asian origin which suggests that Northern pintails are involved in ongoing intercontinental transmission of avian influenza ( Koehler *et al.*, 2008; Ramey *et al.*, 2010). The completion of the genome sequence of species such as the Northern pintail may contribute to a better understanding of disease dynamics and host factors.

The Northern pintail is listed on the IUCN Red List as “least concern” although its population numbers are decreasing ( BirdLife International, 2019). It is Amber-listed in the UK Birds of conservation concern, due to reduced breeding numbers and range ( Stanbury *et al.*, 2021). 

## Genome sequence report

**Figure 1.**
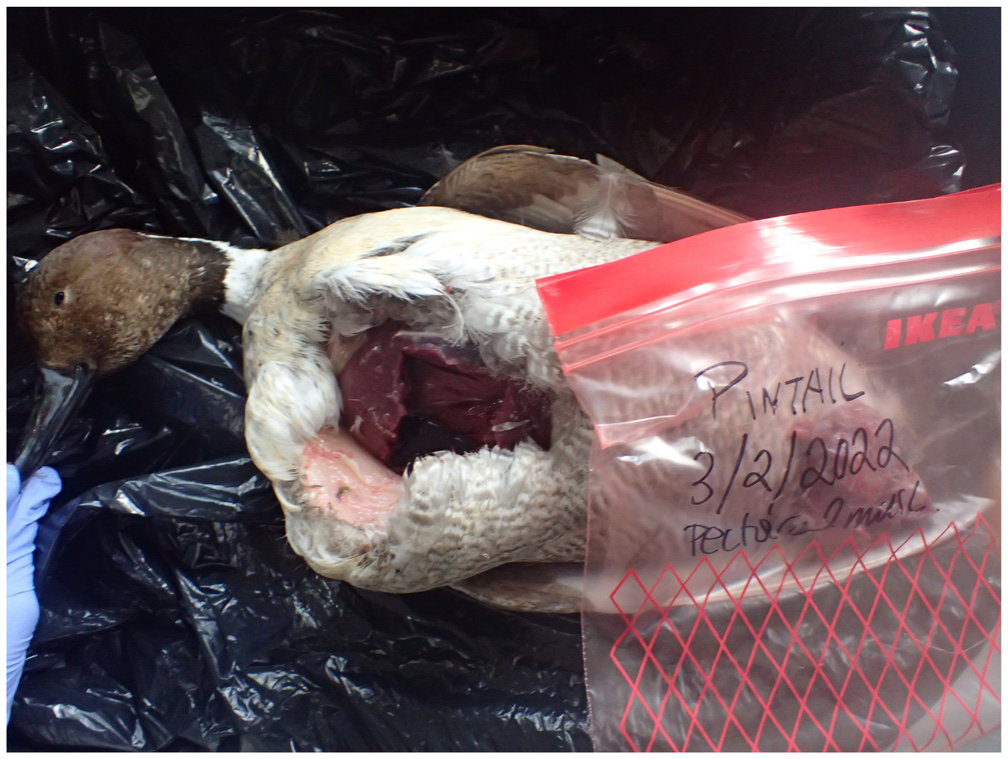
Photograph of the *Anas acuta* (bAnaAcu1) specimen used for genome sequencing.

The genome of an adult *Anas acuta* ( Figure 1) was sequenced using Pacific Biosciences single-molecule HiFi long reads, generating a total of 31.62 Gb (gigabases) from 2.69 million reads, providing approximately 28-fold coverage. Primary assembly contigs were scaffolded with chromosome conformation Hi-C data, which produced 53.70 Gbp from 355.66 million reads, yielding an approximate coverage of 45-fold. Specimen and sequencing information is summarised in Table 1.

**Table 1.** Specimen and sequencing data for *Anas acuta*.

Project information			
**Study title**	Anas acuta (northern pintail)		
**Umbrella BioProject**	PRJEB64746		
**Species**	*Anas acuta*		
**BioSample**	SAMEA112468036		
**NCBI taxonomy ID**	28680		
Specimen information			
**Technology**	**ToLID**	**BioSample accession**	**Organism part**
**PacBio long read sequencing**	bAnaAcu1	SAMEA112468080	muscle
**Hi-C sequencing**	bAnaAcu1	SAMEA112468080	muscle
**RNA sequencing**	bAnaAcu1	SAMEA112468080	muscle
Sequencing information			
**Platform**	**Run accession**	**Read count**	**Base count (Gb)**
**Hi-C Illumina NovaSeq 6000**	ERR11814127	3.56e+08	53.7
**PacBio Sequel IIe**	ERR11809154	2.69e+06	31.62
**RNA Illumina NovaSeq 6000**	ERR12321231	6.90e+07	10.42

**Figure 2.**
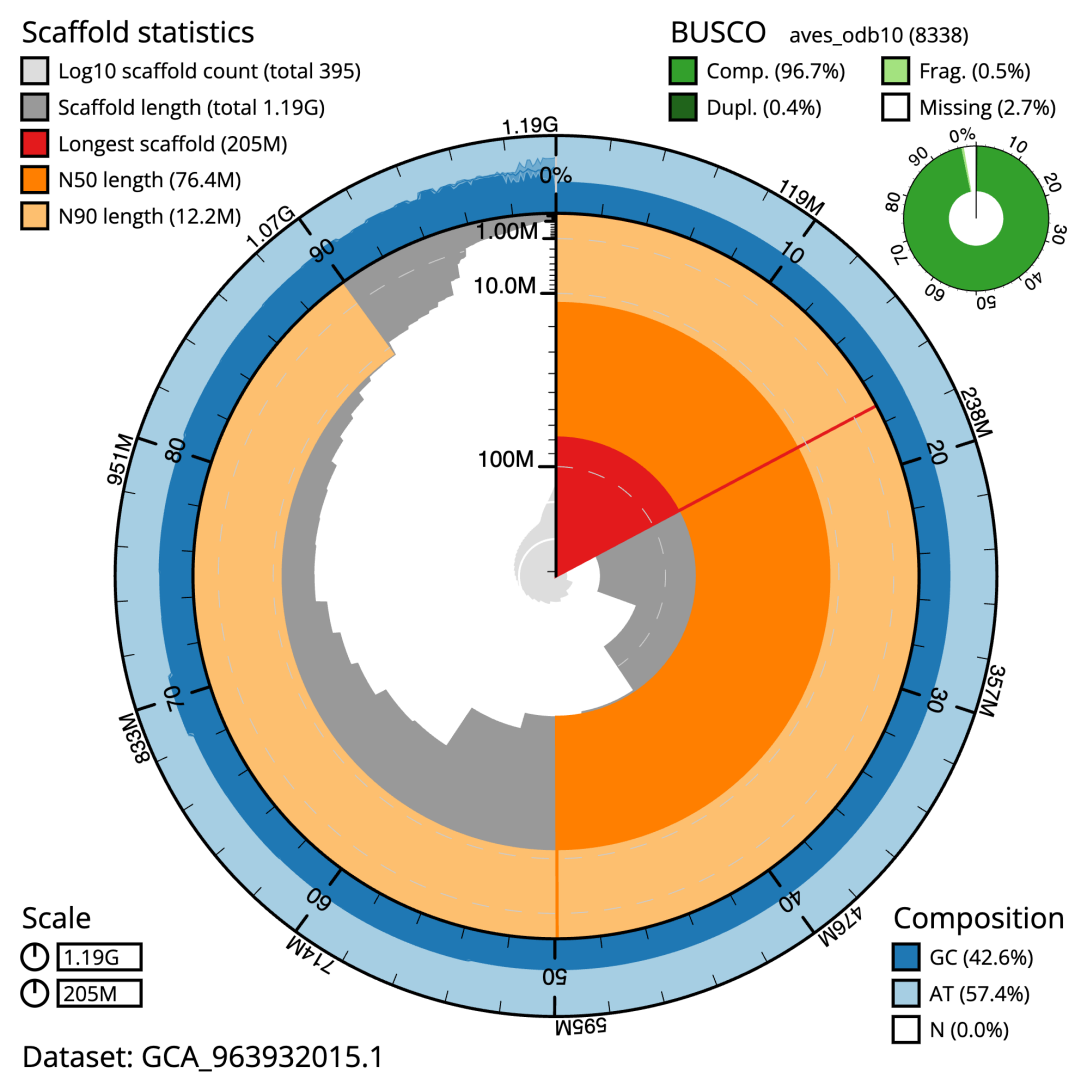
Genome assembly of *Anas acuta*, bAnaAcu1.1: metrics. The BlobToolKit snail plot shows N50 metrics and BUSCO gene completeness. The main plot is divided into 1,000 size-ordered bins around the circumference with each bin representing 0.1% of the 1,189,313,589 bp assembly. The distribution of scaffold lengths is shown in dark grey with the plot radius scaled to the longest scaffold present in the assembly (205,008,147 bp, shown in red). Orange and pale-orange arcs show the N50 and N90 scaffold lengths (76,435,830 and 12,226,428 bp), respectively. The pale grey spiral shows the cumulative scaffold count on a log scale with white scale lines showing successive orders of magnitude. The blue and pale-blue area around the outside of the plot shows the distribution of GC, AT and N percentages in the same bins as the inner plot. A summary of complete, fragmented, duplicated and missing BUSCO genes in the aves_odb10 set is shown in the top right. An interactive version of this figure is available at https://blobtoolkit.genomehubs.org/view/Anas_acuta/dataset/GCA_963932015.1/snail.

**Figure 3.**
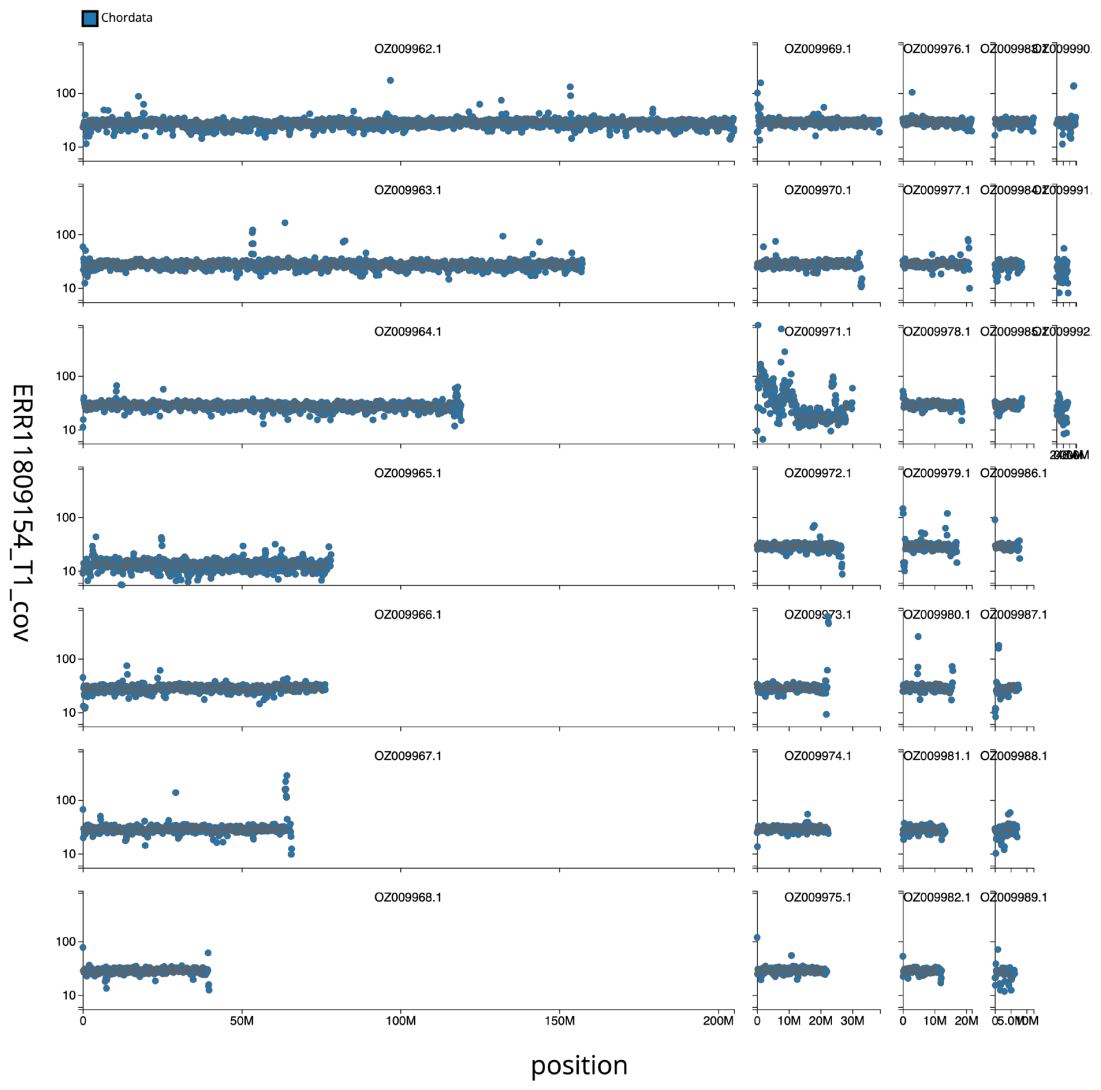
Genome assembly of *Anas acuta*, bAnaAcu1.1: Distribution plot of base coverage in ERR11809154 against position for sequences in assembly GCA_963932015.1. Windows of 1 Mb are coloured by phylum. The assembly has been filtered to exclude sequences with length < 2,550,000. The interactive version can be viewed here.

**Figure 4.**
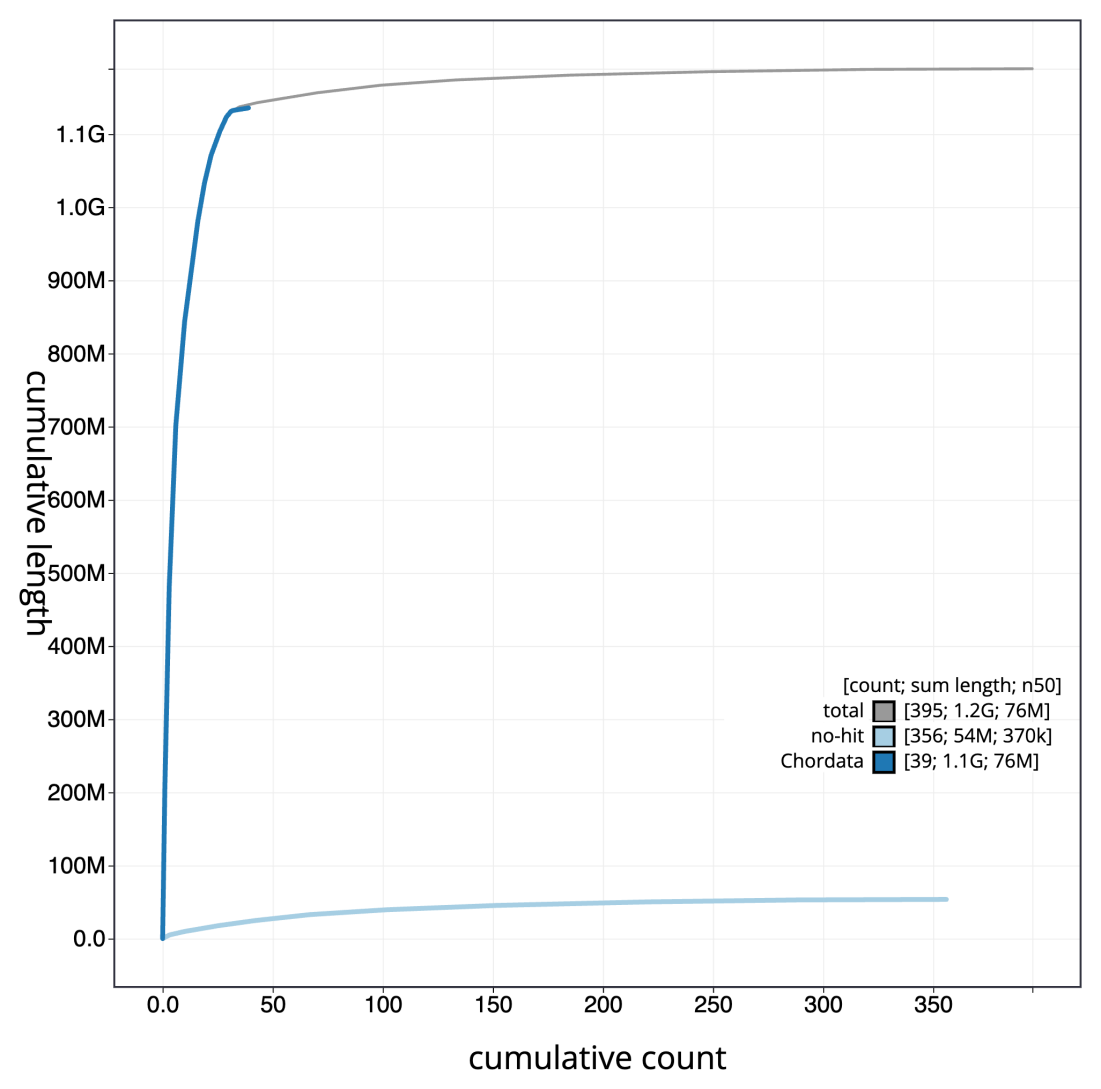
Genome assembly of *Anas acuta* bAnaAcu1.1: BlobToolKit cumulative sequence plot. The grey line shows cumulative length for all sequences. Coloured lines show cumulative lengths of sequences assigned to each phylum using the buscogenes taxrule. An interactive version of this figure is available at https://blobtoolkit.genomehubs.org/view/Anas_acuta/dataset/GCA_963932015.1/cumulative.

**Figure 5.**
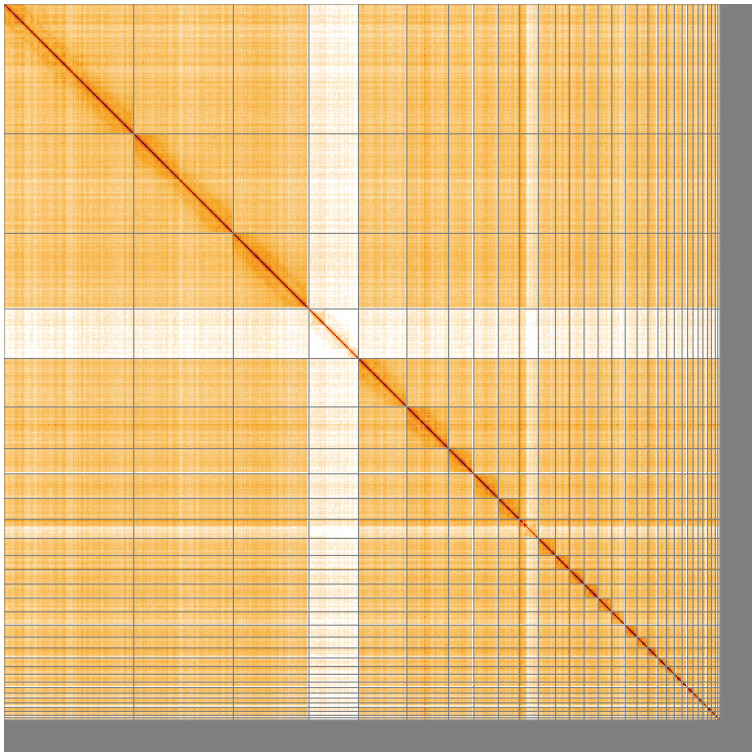
Genome assembly of *Anas acuta* bAnaAcu1.1: Hi-C contact map of the bAnaAcu1.1 assembly, visualised using HiGlass. Chromosomes are shown in order of size from left to right and top to bottom. An interactive version of this figure may be viewed at https://genome-note-higlass.tol.sanger.ac.uk/l/?d=KKAasBSVQ8-hUYw-gBwdkw.

Manual assembly curation corrected 50 missing joins or mis-joins and three haplotypic duplications, reducing the scaffold number by 8.14%. The final assembly has a total length of 1,189.30 Mb in 394 sequence scaffolds with a scaffold N50 of 76.4 Mb ( Table 2). The total count of gaps in the scaffolds is 631. The snail plot in Figure 2 provides a summary of the assembly statistics, while the distribution of base coverage against position per chromosome is shown in Figure 3. The cumulative assembly plot in Figure 4 shows curves for subsets of scaffolds assigned to different phyla. Most (95.79%) of the assembly sequence was assigned to 39 chromosomal-level scaffolds, representing 37 autosomes and the W and Z sex chromosomes. Chromosome-scale scaffolds confirmed by the Hi-C data are named in order of size ( Figure 5; Table 3). The reported karyotype for this species is *n* = 40 ( Abu-Almaaty *et al.*, 2019), however, only 37 autosomes could be confidently assigned during curation. While not fully phased, the assembly deposited is of one haplotype. Contigs corresponding to the second haplotype have also been deposited. The mitochondrial genome was also assembled and can be found as a contig within the multifasta file of the genome submission.

**Table 2.** Genome assembly data for *Anas acuta*, bAnaAcu1.1.

Genome assembly		
Assembly name	bAnaAcu1.1	
Assembly accession	GCA_963932015.1	
*Accession of alternate haplotype*	*GCA_963932075.1*	
Span (Mb)	1,189.30	
Number of contigs	1,026	
Contig N50 length (Mb)	3.4	
Number of scaffolds	394	
Scaffold N50 length (Mb)	76.4	
Longest scaffold (Mb)	205.01	
Assembly metrics *		*Benchmark*
Consensus quality (QV)	58.4	*≥ 50*
*k*-mer completeness	100.0%	*≥ 95%*
BUSCO **	C:97.0%[S:96.5%,D:0.5%], F:0.6%,M:2.4%,n:8,338	*C ≥ 95%*
Percentage of assembly mapped to chromosomes	95.79%	*≥ 95%*
Sex chromosomes	WZ	*localised homologous pairs*
Organelles	Mitochondrial genome: 16.6 kb	*complete single alleles*

* Assembly metric benchmarks are adapted from column VGP-2020 of “Table 1: Proposed standards and metrics for defining genome assembly quality” from Rhie *et al.* (2021). ** BUSCO scores based on the vertebrata_odb10 BUSCO set using version 5.4.3. C = complete [S = single copy, D = duplicated], F = fragmented, M = missing, n = number of orthologues in comparison. A full set of BUSCO scores is available at https://blobtoolkit.genomehubs.org/view/Anas_acuta/dataset/GCA_963932015.1/busco.

**Table 3.** Chromosomal pseudomolecules in the genome assembly of *Anas acuta*, bAnaAcu1.

INSDC accession	Name	Length (Mb)	GC%
OZ009962.1	1	205.01	40.0
OZ009963.1	2	157.35	39.5
OZ009964.1	3	119.29	40.0
OZ009966.1	4	76.44	40.0
OZ009967.1	5	65.85	41.5
OZ009968.1	6	39.89	41.5
OZ009969.1	7	38.68	42.0
OZ009970.1	8	33.12	42.5
OZ009972.1	9	26.99	43.0
OZ009973.1	10	22.62	42.5
OZ009974.1	11	22.53	43.5
OZ009975.1	12	22.09	43.5
OZ009976.1	13	21.75	43.0
OZ009977.1	14	21.13	45.5
OZ009978.1	15	18.73	45.5
OZ009979.1	16	17.16	46.0
OZ009980.1	17	15.88	46.0
OZ009981.1	18	13.46	47.0
OZ009982.1	19	12.38	47.5
OZ009983.1	20	12.23	48.5
OZ009984.1	21	8.64	51.0
OZ009985.1	22	8.61	48.0
OZ009986.1	23	7.93	49.5
OZ009987.1	24	7.69	52.0
OZ009988.1	25	7.16	51.5
OZ009989.1	26	6.34	53.0
OZ009990.1	27	6.15	49.5
OZ009991.1	28	3.76	57.0
OZ009992.1	29	3.64	56.5
OZ009993.1	30	1.88	50.0
OZ009994.1	31	1.59	53.0
OZ009995.1	32	1.5	54.5
OZ009996.1	33	0.57	57.0
OZ009997.1	34	0.43	55.5
OZ009998.1	35	0.37	58.5
OZ009999.1	36	0.22	54.5
OZ010000.1	37	0.12	61.5
OZ009971.1	W	30.11	48.0
OZ009965.1	Z	78.26	39.5
OZ010001.1	MT	0.02	49.0

The estimated Quality Value (QV) of the final assembly is 58.4 with *k*-mer completeness of 100.0%, and the assembly has a BUSCO v5.4.3 completeness of 97.0% (single = 96.5%, duplicated = 0.5%), using the vertebrata_odb10 reference set ( *n* = 8,338).

Metadata for specimens, BOLD barcode results, spectra estimates, sequencing runs, contaminants and pre-curation assembly statistics are given at https://tolqc.cog.sanger.ac.uk/darwin/birds/Anas_acuta/.

## Methods

### Sample acquisition

Several small samples of pectoral muscle were taken from a wild specimen (specimen ID NHMUK014561648, ToLID bAnaAcu1) found deceased and collected at WWT Slimbridge, Gloucestershire in 2022 as part of a disease surveillance programme carried out by WWT in contribution to the Great Britain Wildlife Health Partnership. The specimen had the appearance of a male, but post-mortem dissection showed that it was female. The samples were preserved at –80 °C. The specimen was collected and identified by Michelle O'Brien (Wildfowl & Wetlands Trust).

### Nucleic acid extraction

The workflow for high molecular weight (HMW) DNA extraction at the Wellcome Sanger Institute (WSI) Tree of Life Core Laboratory includes a sequence of core procedures: sample preparation; sample homogenisation, DNA extraction, fragmentation, and clean-up. In sample preparation, the bAnaAcu1 sample was weighed and dissected on dry ice ( Jay *et al.*, 2023). For sample homogenisation, muscle tissue was cryogenically disrupted using the Covaris cryoPREP ^®^ Automated Dry Pulverizer ( Narváez-Gómez *et al.*, 2023).

HMW DNA was extracted at the WSI Scientific Operations core using the Automated MagAttract v2 protocol ( Oatley *et al.*, 2023). The DNA was sheared into an average fragment size of 12–20 kb in a Megaruptor 3 system ( Bates *et al.*, 2023). Sheared DNA was purified by solid-phase reversible immobilisation ( Strickland *et al.*, 2023): in brief, the method employs AMPure PB beads to eliminate shorter fragments and concentrate the DNA. The concentration of the sheared and purified DNA was assessed using a Nanodrop spectrophotometer and Qubit Fluorometer using the Qubit dsDNA High Sensitivity Assay kit. Fragment size distribution was evaluated by running the sample on the FemtoPulse system.

RNA was extracted from muscle tissue of bAnaAcu1 in the Tree of Life Laboratory at the WSI using the RNA Extraction: Automated MagMax™ *mir*Vana protocol ( do Amaral *et al.*, 2023). The RNA concentration was assessed using a Nanodrop spectrophotometer and a Qubit Fluorometer using the Qubit RNA Broad-Range Assay kit. Analysis of the integrity of the RNA was done using the Agilent RNA 6000 Pico Kit and Eukaryotic Total RNA assay.

Protocols developed by the WSI Tree of Life laboratory are publicly available on protocols.io ( Denton *et al.*, 2023).

### Sequencing

Pacific Biosciences HiFi circular consensus DNA sequencing libraries were constructed according to the manufacturers’ instructions. Poly(A) RNA-Seq libraries were constructed using the NEB Ultra II RNA Library Prep kit. DNA and RNA sequencing was performed by the Scientific Operations core at the WSI on Pacific Biosciences Sequel IIe (HiFi) and Illumina NovaSeq 6000 (RNA-Seq) instruments. Hi-C data were also generated from muscle tissue of bAnaAcu1 using the Arima-HiC v2 kit. The Hi-C sequencing was performed using paired-end sequencing with a read length of 150 bp on the Illumina NovaSeq 6000 instrument.

### Genome assembly, curation and evaluation

***Assembly***

The original assembly of HiFi reads was performed using Hifiasm ( Cheng *et al.*, 2021) with the --primary option. Haplotypic duplications were identified and removed with purge_dups ( Guan *et al.*, 2020). Hi-C reads are further mapped with bwa-mem2 ( Vasimuddin *et al.*, 2019) to the primary contigs, which are further scaffolded using the provided Hi-C data ( Rao *et al.*, 2014) in YaHS ( Zhou *et al.*, 2023) using the --break option. Scaffolded assemblies are evaluated using Gfastats ( Formenti *et al.*, 2022), BUSCO ( Manni *et al.*, 2021) and MERQURY.FK ( Rhie *et al.*, 2020).

The mitochondrial genome was assembled using MitoHiFi ( Uliano-Silva *et al.*, 2023), which runs MitoFinder ( Allio *et al.*, 2020) and uses these annotations to select the final mitochondrial contig and to ensure the general quality of the sequence.

***Assembly curation***

The assembly was decontaminated using the Assembly Screen for Cobionts and Contaminants (ASCC) pipeline (article in preparation). Flat files and maps used in curation were generated in TreeVal ( Pointon *et al.*, 2023). Manual curation was primarily conducted using PretextView ( Harry, 2022), with additional insights provided by JBrowse2 ( Diesh *et al.*, 2023) and HiGlass ( Kerpedjiev *et al.*, 2018). Scaffolds were visually inspected and corrected as described by Howe *et al*. (2021). Any identified contamination, missed joins, and mis-joins were corrected, and duplicate sequences were tagged and removed. The sex chromosomes were assigned based on read coverage statistics. The entire process is documented at https://gitlab.com/wtsi-grit/rapid-curation (article in preparation).

***Evaluation of the final assembly***

The final assembly was post-processed and evaluated with the three Nextflow ( Di Tommaso *et al.*, 2017) DSL2 pipelines “sanger-tol/readmapping” ( Surana *et al.*, 2023a), “sanger-tol/genomenote” ( Surana *et al.*, 2023b), and “sanger-tol/blobtoolkit” ( Muffato *et al.*, 2024). The pipeline sanger-tol/readmapping aligns the Hi-C reads with bwa-mem2 ( Vasimuddin *et al.*, 2019) and combines the alignment files with SAMtools ( Danecek *et al.*, 2021). The sanger-tol/genomenote pipeline transforms the Hi-C alignments into a contact map with BEDTools ( Quinlan & Hall, 2010) and the Cooler tool suite ( Abdennur & Mirny, 2020), which is then visualised with HiGlass ( Kerpedjiev *et al.*, 2018). It also provides statistics about the assembly with the NCBI datasets ( Sayers *et al.*, 2024) report, computes *k*-mer completeness and QV consensus quality values with FastK and MERQURY.FK, and a completeness assessment with BUSCO ( Manni *et al.*, 2021).

The sanger-tol/blobtoolkit pipeline is a Nextflow port of the previous Snakemake Blobtoolkit pipeline ( Challis *et al.*, 2020). It aligns the PacBio reads with SAMtools and minimap2 ( Li, 2018) and generates coverage tracks for regions of fixed size. In parallel, it queries the GoaT database ( Challis *et al.*, 2023) to identify all matching BUSCO lineages to run BUSCO ( Manni *et al.*, 2021). For the three domain-level BUSCO lineage, the pipeline aligns the BUSCO genes to the Uniprot Reference Proteomes database ( Bateman *et al.*, 2023) with DIAMOND ( Buchfink *et al.*, 2021) blastp. The genome is also split into chunks according to the density of the BUSCO genes from the closest taxonomically lineage, and each chunk is aligned to the Uniprot Reference Proteomes database with DIAMOND blastx. Genome sequences that have no hit are then chunked with seqtk and aligned to the NT database with blastn ( Altschul *et al.*, 1990). All those outputs are combined with the blobtools suite into a blobdir for visualisation.

The genome assembly and evaluation pipelines were developed using the nf-core tooling ( Ewels *et al.*, 2020), use MultiQC ( Ewels *et al.*, 2016), and make extensive use of the Conda package manager, the Bioconda initiative ( Grüning *et al.*, 2018), the Biocontainers infrastructure ( da Veiga Leprevost *et al.*, 2017), and the Docker ( Merkel, 2014) and Singularity ( Kurtzer *et al.*, 2017) containerisation solutions.

Table 4 contains a list of relevant software tool versions and sources.

**Table 4.** Software tools: versions and sources.

Software tool	Version	Source
BEDTools	2.30.0	https://github.com/arq5x/bedtools2
BLAST	2.14.0	ftp://ftp.ncbi.nlm.nih.gov/blast/executables/blast+/
BlobToolKit	4.3.7	https://github.com/blobtoolkit/blobtoolkit
BUSCO	5.4.3 and 5.5.0	https://gitlab.com/ezlab/busco
bwa-mem2	2.2.1	https://github.com/bwa-mem2/bwa-mem2
Cooler	0.8.11	https://github.com/open2c/cooler
DIAMOND	2.1.8	https://github.com/bbuchfink/diamond
fasta_windows	0.2.4	https://github.com/tolkit/fasta_windows
FastK	427104ea91c78c3b8b8b49f1a7d6bbeaa869ba1c	https://github.com/thegenemyers/FASTK
Gfastats	1.3.6	https://github.com/vgl-hub/gfastats
GoaT CLI	0.2.5	https://github.com/genomehubs/goat-cli
Hifiasm	0.19.5-r587	https://github.com/chhylp123/hifiasm
HiGlass	44086069ee7d4d3f6f3f0012569789ec138f42b84a a44357826c0b6753eb28de	https://github.com/higlass/higlass
Merqury.FK	d00d98157618f4e8d1a9190026b19b471055b22e	https://github.com/thegenemyers/MERQURY.FK
MitoHiFi	3	https://github.com/marcelauliano/MitoHiFi
MultiQC	1.14, 1.17, and 1.18	https://github.com/MultiQC/MultiQC
NCBI Datasets	15.12.0	https://github.com/ncbi/datasets
Nextflow	23.04.0-5857	https://github.com/nextflow-io/nextflow
PretextView	0.2	https://github.com/sanger-tol/PretextView
purge_dups	1.2.5	https://github.com/dfguan/purge_dups
samtools	1.16.1, 1.17, and 1.18	https://github.com/samtools/samtools
sanger-tol/ascc	-	https://github.com/sanger-tol/ascc
sanger-tol/ genomenote	1.1.1	https://github.com/sanger-tol/genomenote
sanger-tol/ readmapping	1.2.1	https://github.com/sanger-tol/readmapping
Seqtk	1.3	https://github.com/lh3/seqtk
Singularity	3.9.0	https://github.com/sylabs/singularity
TreeVal	1.0.0	https://github.com/sanger-tol/treeval
YaHS	1.1a.2	https://github.com/c-zhou/yahs

### Wellcome Sanger Institute – Legal and Governance

The materials that have contributed to this genome note have been supplied by a Darwin Tree of Life Partner. The submission of materials by a Darwin Tree of Life Partner is subject to the **‘Darwin Tree of Life Project Sampling Code of Practice’**, which can be found in full on the Darwin Tree of Life website here. By agreeing with and signing up to the Sampling Code of Practice, the Darwin Tree of Life Partner agrees they will meet the legal and ethical requirements and standards set out within this document in respect of all samples acquired for, and supplied to, the Darwin Tree of Life Project. 

Further, the Wellcome Sanger Institute employs a process whereby due diligence is carried out proportionate to the nature of the materials themselves, and the circumstances under which they have been/are to be collected and provided for use. The purpose of this is to address and mitigate any potential legal and/or ethical implications of receipt and use of the materials as part of the research project, and to ensure that in doing so we align with best practice wherever possible. The overarching areas of consideration are:

•     Ethical review of provenance and sourcing of the material

•     Legality of collection, transfer and use (national and international) 

Each transfer of samples is further undertaken according to a Research Collaboration Agreement or Material Transfer Agreement entered into by the Darwin Tree of Life Partner, Genome Research Limited (operating as the Wellcome Sanger Institute), and in some circumstances other Darwin Tree of Life collaborators.

## Data Availability

European Nucleotide Archive: *Anas acuta* (northern pintail). Accession number PRJEB64746; https://identifiers.org/ena.embl/PRJEB64746 ( Wellcome Sanger Institute, 2024). The genome sequence is released openly for reuse. The *Anas acuta* genome sequencing initiative is part of the Darwin Tree of Life (DToL) project. All raw sequence data and the assembly have been deposited in INSDC databases. The genome will be annotated using available RNA-Seq data and presented through the Ensembl pipeline at the European Bioinformatics Institute. Raw data and assembly accession identifiers are reported in Table 1 and Table 2.

## Author information

Members of the Natural History Museum Genome Acquisition Lab are listed here: https://doi.org/10.5281/zenodo.12159242.

Members of the Darwin Tree of Life Barcoding collective are listed here: https://doi.org/10.5281/zenodo.12158331

Members of the Wellcome Sanger Institute Tree of Life Management, Samples and Laboratory team are listed here: https://doi.org/10.5281/zenodo.12162482.

Members of Wellcome Sanger Institute Scientific Operations: Sequencing Operations are listed here: https://doi.org/10.5281/zenodo.12165051.

Members of the Wellcome Sanger Institute Tree of Life Core Informatics team are listed here: https://doi.org/10.5281/zenodo.12160324.

Members of the Tree of Life Core Informatics collective are listed here: https://doi.org/10.5281/zenodo.12205391.

Members of the Darwin Tree of Life Consortium are listed here: https://doi.org/10.5281/zenodo.4783558.
