# Supplementary material for: The genome sequence of the northern pintail, Anas acuta Linnaeus, 1758
Source: Wellcome Open Res. 2024 Aug 12;9:446. [Version 1] doi: 10.12688/wellcomeopenres.22770.1 (PMC12464533; doi:10.12688/wellcomeopenres.22770.1)
Supplement: Peer review history

# Reviewer report: Reviewer response for version 1

**DOI:** 10.21956/wellcomeopenres.25074.r128919

**Published:** 2025-09-25

**Authors:** Annabel Whibley

**Affiliations:** 1 Grapevine Improvement, Bragato Research Institute, Lincoln, New Zealand; 2 The University of Auckland, Auckland, New Zealand

O’Brien and colleagues report the genome assembly of *Anas acuta*, the Northern Pintail duck. A female individual was sampled, and so both Z and W sex chromosomes are recovered. The natural history of the organism is set out nicely in the introduction and indicates that there is a sex difference in plumage. Interestingly, the sample used for this reference genome  was initially identified as male but found, upon dissection, to be female.

 The data input to assembly follows the DToL standard of HiFi backbone (approx. 30x coverage), and a scaffolding Illumina Hi-C dataset (approx. 45x coverage). An Illumina RNAseq dataset was also generated from muscle tissue from the same sample, but an annotation is not reported in this Data Note. The resulting assembly is of high quality and follows bioinformatics and reporting templates. Metadata relating to the sample and software tools and the assembly evaluation metrics are comprehensively reported. Data accessions are active.

 The inclusion of a coverage plot in 1Mb windows along the chromosomes is a visualization I have not previously encountered in this report type, and which I welcome. I wonder if there could be improvements that make it easier still to read. The relatively poor assembly/challenge of the beginning of the W chromosome, and some of the micro-chromosomes is apparent in these plots and is really useful headline information for genome “users”, but the difference between n and 2n coverage levels, which distinguishes the sex chromosomes from the autosomes is there but takes a careful look to pick out- perhaps adding a line showing the median genome coverage might help (for most chromosomes it would be entirely masked by the actual datapoints but it would help to highlight coverage anomalies.

Are sufficient details of methods and materials provided to allow replication by others?

Yes

Is the rationale for creating the dataset(s) clearly described?

Yes

Are the datasets clearly presented in a useable and accessible format?

Yes

Are the protocols appropriate and is the work technically sound?

Yes

Reviewer Expertise:

Genomics, bioinformatics

I confirm that I have read this submission and believe that I have an appropriate level of expertise to confirm that it is of an acceptable scientific standard.

# Reviewer report: Reviewer response for version 1

**DOI:** 10.21956/wellcomeopenres.25074.r128913

**Published:** 2025-09-15

**Authors:** Merly Escalona

**Affiliations:** 1 University of California, Santa Cruz, USA

The manuscript discusses the development of genomic resources for the northern pintail (Anas acuta), a species considered of least concern but with declining population numbers and listed as Amber in the UK Birds of Conservation Concern. These resources include a new chromosome-level genome assembly, a mitochondrial genome, and the corresponding annotations for both. The genome was assembled using PacBio HiFi long reads and scaffolded with Hi-C data, followed by manual curation. The methods for data generation, processing, assembly, annotation, and quality checks are thoroughly documented and described mostly well. The metadata provided is comprehensive. The generated resources are high-quality, and the assembly metrics meet the general benchmarks for quality, k-mer completeness, and BUSCO scores, as established by the Vertebrate Genome Project (Rhie et al. 2021) and the Earth BioGenome Project (https://www.earthbiogenome.org/report-on-assembly-standards).

 Overall, it is a solid manuscript that meets the Journal's submission requirements. However, there are a few aspects to consider. See comments below.

 - Although it seems clear that the main justification for the manuscript and resources is simply the creation and availability of the resources, it would be better to be more explicit in the manuscript.

 The manuscript describes the genome assembly of Anas acuta. However, the authors generate a diploid representation following the primary/alternate strategy and naming convention. I recommend sharing the metrics of the alternate assembly and the primary one as a supplementary table, even if the alternate assembly isn't at the same quality level as the primary. You are generating both assemblies, and both are being made publicly available.

 - Given the above, how are you achieving a genome with 100% k-mer completeness? Are these species 100% homozygous? If not, are you separating k-mers based on haplotype? How is that done if the assemblies are only partially phased? Or is the k-mer completeness calculated from the diploid assembly?

 - Although both the nuclear and mitochondrial genomes have been assembled, annotated, and made publicly available, there are no metrics available to assess the quality of the mitochondrial genome, and it would be helpful to share those metrics as well.

Are sufficient details of methods and materials provided to allow replication by others?

Yes

Is the rationale for creating the dataset(s) clearly described?

Partly

Are the datasets clearly presented in a useable and accessible format?

Yes

Are the protocols appropriate and is the work technically sound?

Yes

Reviewer Expertise:

genome assembly, bioinformatics

I confirm that I have read this submission and believe that I have an appropriate level of expertise to confirm that it is of an acceptable scientific standard.
